# Peer education model in Trakya university faculty of medicine

**DOI:** 10.1186/s12909-023-04739-7

**Published:** 2023-10-06

**Authors:** Nermin Sakru, Feza Irem Aldi, Berrak Cakmakci, Furkan Arabaci, Erkut Afyoncu

**Affiliations:** Department of Medical Microbiology, Faculty of Medicine, Trakya University, Edirne, Turkey

**Keywords:** Peer assisted learning, Peer education, Medical school, Parasitology, Turkey

## Abstract

**Background:**

Peer education is an education model applied to change knowledge, behavior, and attitude in groups equal to each other regarding age, education, and status. This model is preferred in universities to improve teaching skills and reduce the stress level of students. This study aims to apply the peer education model at Trakya University Faculty of Medicine to receive feedback from students and to examine its effect on exam results.

**Methods:**

This cross-sectional, descriptive, and analytical study was conducted with second-year medical students in parasitology laboratory lessons. Eighteen out of a total of 264 students were selected as peer educators. Peer educators have reached the level of providing education to students by taking the training before the laboratory lessons. At the end of the study, questionnaires were applied to peer educators and students. The students’ of 2021–2022 exam results were compared with the 2018–2019 academic year results.

**Results:**

A total of 233 students were surveyed, and 78.5% (183/233) of them believe peer education is helpful, 69.9% (163/233) think it can help them reinforce what they have learned in theoretical lessons, 54.5% (127/233) think it should be used in other practical lessons, and 64.3% think it should be used in the coming years. While there was no significant difference between the exam results of the students in 2021–2022 and the 2018–2019 period (p: 0.462), a significant difference was found between the exam results of peer educators and students (p < 0.05).

**Conclusions:**

It is known that with the peer education model, student stress will decrease, and interest and participation in the lesson will increase. Continuing this education model in the coming years and expanding it to other laboratory courses will benefit medical education.

## Background

Peer education is an education model used to change and improve attitudes, behaviors, and knowledge in groups interacting socially and having similar attitudes and behaviors regarding age and educational status. The definition of peer education as “people from similar social groups who help each other learn and learn by teaching themselves without a professional tutor” is also widely used [1]. In the literature, peer education studies are expressed in various ways, such as peer teaching, peer-assisted learning, and peer counseling [2–5].

Pedagogical origins of peer education are based on scientists such as Piaget and Perry. This education model was first used in the United States in the 1960s to inform and educate students about harmful habits such as committing crimes and alcohol and drug use [6]. From this moment on, peer education began to be used gradually, and in 1991, the World Health Organization (WHO) decided to initiate worldwide studies to prevent the spread and transmission of various diseases, especially HIV/AIDS. As a result, in 1993, health education authorities prepared a peer education program at the University of Manchester to prevent the spread of AIDS among adults [7]. Today, peer education is used in many fields, such as schools, hospitals, workplaces, and various subjects [2, 4, 8–10]. The European Peer Training Organization, EPTO, headquartered in Belgium, has been working on issues such as the development of individuals, motivation, and self-confidence since 2007, helping young people get to know their own culture and discover how they can lead a better life [11]. In our country, in the joint study titled “Turkey’s Peer Education Project Against Addiction” conducted by the Ministry of Youth and Sports with the Green Crescent [12], in the “Social Cohesion and Adaptation Process and Child Rights Trainings” conducted by the Ministry of Family and Social Policies in cooperation with UNICEF, peer education model has started to be implemented [13].

Peer education is an alternative method to improve learning skills before and after graduation in medical faculties [8, 14–17]. In a study conducted by Soriano et al. in 2010, it was shown that 99 (76%) out of 130 medical schools in the United States used the peer education model during medical education [18]. This education model was used in various medical faculties in our country, and the results were shared [19–21]. Studies show that peer education in medical faculties benefits students, faculty members, and even faculties [2, 8, 14, 19–24].

This study aims to apply the peer education model in Trakya University Medical Faculty Parasitology practices and to evaluate the students’ views on this subject and exam results. Our study will set an example in our country regarding the application of peer education in the medical faculty and contribute to the literature.

## Methods

This study was approved by the Trakya University Faculty of Medicine Non-Interventional Scientific Research Ethics Committee with protocol number TÜTF-GOBAEK 2022/60.

This research is a cross-sectional, descriptive, and analytical study. It was conducted in 6 parasitology laboratory lessons in Trakya University Faculty of Medicine 2021–2022 academic year 2nd -grade 8th course committee (Introduction to Clinical Sciences and Pathology Board).

“Introduction to Clinical Sciences and Pathology Board” is a board with main courses such as pharmacology, pathology and microbiology. This board consists of 13 laboratory lessons. Parasitology practices have an important place in laboratory courses, of which 6 are microbiology and 6 are parasitology laboratory courses.

### Selection and training of peer educators

Peer educators were selected by systematic random sampling from 264 students who will attend 2nd grade classes for the first time, and 18 of those selected volunteered to be peer educators. The study was applied to the entire student group, no sampling was done. While calculating the number of peer educators, the calculation was made according to the number of tables in the laboratory lesson. The training of peer educators was given by parasitology lecturers before the laboratory lesson, and it was ensured that the educators reached the level of providing education to the students. All students were informed about the study in the first laboratory lesson, and peer educators were introduced. In the laboratory lessons, the lecturers responsible were also present with the peer educators.

### Data collection

At the end of the study, the exam results were evaluated. Since face-to-face education could not be provided in the 2019–2020 and 2020–2021 academic years due to the Covid-19 pandemic, the exam results of the 2018–2019 academic year 2nd -grade students were used to compare the 2021–2022 data.

Two separate questionnaires were prepared and applied to peer educators and students. The questionnaire forms used 5-point Likert-type questions and one open-ended question. While the questionnaire applied to the peer educators consists of 7 questions, the questionnaire applied to the students consists of 14 questions. We have provided feedback from students for evaluation of our study. The feedback included basic information, learning attitude, participation, interpersonal relationship, and organizational approach. Our feedback questions were preliminary tested on 3rd grade students.

### Statistical analysis

The collected data were analyzed using SPSS 22 statistical analysis program. Number, percentage, arithmetic mean, standard deviation, and standard error were used to evaluate descriptive statistics. Independent T-test and Mann-Whitney U test were used when comparing the groups’ test scores. A value of p < 0.05 was considered statistically significant.

## Results

The study was conducted with 264 Trakya University Faculty of Medicine 2nd year students. All 18 peer educators and 88.3% (233/246) students participated in the questionnaires.

### Student questionnaire findings

According to the survey results of two hundred and thirty-three students, 97.0% (226/233) of the students stated that they did not hesitate when asking a question to the peer educator, 90.1% (210/233) to the research assistant, 71.7% (167/233) to the lecturer. 78.5% (183/233) of the students stated that peer education was beneficial in practice, and 69.9% (163/233) stated that it enabled them to reinforce what they learned in theoretical lessons with laboratory and clinical skills training. 54.5% (127/233) of the students stated that they recommend the peer education applied in the implementation of other courses, and 64.3% (150/233) in the following years.

According to the survey in which eighteen peer educators participated, 100.0% (18/18) stated that being a peer tutor was beneficial in practice, increased their interest in the course, and provided the opportunity to participate actively in laboratory and clinical skills training. 88.9% (16/18) of them stated that they wanted to be a peer educator in the practice of other courses, this enabled them to reinforce what they learned in theoretical courses with laboratory and clinical skills training, and they wanted to be a peer educator again.

### Exam results findings

We analyzed and compared scores of; (a) the end-of-year exam (b) the 8th board exam, (c) 8th board laboratory exam that included parasitology and other:

In the 2018–2019 academic year, (a) the mean of the students’ end-of-year exam scores was 64.02 out of 100 points (± SE:0.396), SD:6.45; in the 2021–2022 academic year, the mean of the theoretical exam scores of the students (excluding peer educators) was 61.15 (± SE: 0.791), SD: 12.85, and there was no significant difference between the means of both groups (p: 0.462). While the mean score of the (b) 8th board of the 2018–2019 student group was 58.53 (± SE:0.566) out of 100 points, this value was 58.67 (± SE:0.718) for the 2021–2022 student group (excluding peer educators), and no significant difference was found between the mean score of the two groups (p:0.873). While the mean score of the (c) 8th board laboratory exam for the 2018–2019 student group was 76.06 (± SE:0.701) out of 100 points, this value was 76.54 (± SE:0.835) for the 2021–2022 student group (excluding peer educators), and no significant difference was found between the mean score of both groups (p:0.653).

While the mean parasitology laboratory exam score of 279 students in the 2018–2019 academic year was 34.17 (± SE:0.52) out of 50 points, SD:8.68; in 2021–2022, parasitology laboratory exam mean score of 241 students (peer educators and those who did not take the exam were excluded) was 33.78 (± SE: 0.59), SD: 9.23, and no significant difference was found between the mean score of both groups (p = 0.621). The mean parasitology practice scores of the 2021–2022 student group and peer educators are 33.78 (± SE:0.595) for the student group, 43.22 (± SE:1.225) for the peer educator group out of 50 points, and a significant difference was found between the students and peer educators (p˂0.05).

The mean score of the 8th board, excluding parasitology, for the 2021–2022 student group and the peer educators was 42.76 (± SE:0.397) for the student group, 43.33 (± SE:1.309) for the peer educator group, out of 50 points, and there was no significant difference between the mean score of both groups (p:0.689).

## Discussion

In classical education methods, hierarchical differences and power imbalance problems are seen compared to the peer education model. In the peer education model, the problems mentioned in classical education methods do not arise since people who are similar in age, education, and status train each other. In addition, being more accepted by students as a result of receiving education from their peers, understanding what they have experienced during education, and personal identification are important advantages in reducing students’ anxiety. Various studies have reported that students can ask questions to their peers more easily, thus reducing their anxiety and stress [8, 21, 25–29]. In the study of Talapatra et al. [8] with medical students, the satisfaction level of the students with the peer education model was 4.96 out of 5, and they reported that they could easily ask questions to peer educators when necessary. A study by Şancı et al. [25] with nursing students concluded that students talked about course subjects more easily with peer educators, and their anxiety decreased. In the study conducted by Aydın et al. [21] in the questionnaire where 5 was the highest score, the students stated that the peer education environment is a more comfortable environment for asking questions and gave the application a score of 4.18. In our study, 97% of the students (226/233) reported that they did not hesitate when asking questions to the peer educator. This rate decreased to 71.7% (167/233) when asking questions to the lecturer. Our results are consistent with the studies in the literature [8, 21, 25] and reveal that peer education creates a more comfortable learning environment.

In the classical education model, the one-way information flow from the educator to the student continues interactively in the peer education model. The absence of hierarchical differences among peers and the fact that they use similar language create a more suitable learning environment by eliminating the concepts of reward or punishment [30, 31]. Gök [32], Qin et al. [2] observed in their studies that peer education increased interest and participation in the lesson. Yang and Wang [33], who created a peer learning and assessment model (PLAM) and evaluated students’ feedback, also reported that the method increased students’ interest in learning and improved their abilities. In our study, following the results of the literature [2, 32, 33], 78.5% of the students stated that peer education was beneficial in practice, 65.6% (153/233) stated that it provided the opportunity to actively participate in laboratory and clinical skills, 69.9% (163/233) stated that it enabled them to reinforce what they learned in theoretical courses with laboratory and clinical skills training, and 47.7% of them stated that it increased their interest in the course. In addition, 64.3% (150/233) of the students suggested peer education be implemented in the coming years.

Peer education practices are very few in medical faculties in our country. Özan and Yurdabakan [19] conducted a study in which they examined the effects of self- and peer-assessment practice on basic communication skills in medical students. Musal [20] implemented a peer counseling program so that term 1 students would not experience adjustment difficulties and shared two-year results. Aydın et al. [21] applied phlebotomy training to term 3 students by specialist students and peer educators and evaluated the students’ opinions. Our study can be considered a first in our country in terms of both evaluating student views and examining the effect on exam success.

In studies where the effectiveness of the peer education system is evaluated, most of the data is based on the feedback of the participants [8, 23, 25, 34–36]. The number of studies focusing on objective results, such as the effect of peer education on exam results, is less [37–42]. The results of studies on the effect of peer education on exam results also show differences. Manyama et al. [39] found the exam performances of students after peer education to be significantly higher than their exam performances in the traditional education method in their study, in which they compared the traditional education method and peer education in anatomy lessons. On the other hand, Zarifnejad et al. [40] reported that no statistically significant improvement was achieved in the exam results in their study. In our study, no statistically significant difference was found between the exam means in the education year in which the peer education model was not applied and the education year in which the peer education model was applied. We expected that the student group receiving peer education would have a higher exam performance. We think that Covid-19 had an impact on our results. The Covid-19 epidemic has affected the education system in our country as well as all over the world. With the transition to distance education, medical education, which has laboratory courses, bedside practices, and case/patient presentations, has been interrupted to some extent [36, 43–45]. The students with whom we applied peer education were 2nd -year students studying distance education due to the epidemic and started university life after two years. Therefore, the lack of significant difference between exam results may be related to the difficulty of adapting of students to the education system.

It has long been suggested that learning a subject by teaching contributes to developing a deeper and improved understanding of the subject and is easier to remember [1, 46]. According to the EPTO, peer education encourages young people to find solutions to their own problems, to shape their own lives and the world around them [11]. In our study, peer educators stated that they were interested in being an educator and that they might consider pursuing an academic career in the future. A peer trainer said, “I learned in this study that learning by teaching is so memorable and enjoyable. It was a project that I will never forget throughout my education life and that has an important place in my social and academic life.” Peer trainers improved their social skills, leadership qualities and academic aspects with this project. When we look at the results of the questionnaires made to the peer educators in our study, all of the peer educators (18/18) stated that they thought that being a peer educator was beneficial in practice, increased their interest in the lesson, and provided the opportunity to participate in laboratory and clinical skills actively. 88.9% (16/18) of the peer educators stated that they want to be a peer tutor in the practices of other courses. Peer education has subjective results, such as student satisfaction, participation, and learning, and objective results, such as academic success [37–39, 41]. In their study [37], in which Gregory et al. examined the effects of peer educators on learning outcomes, they found the mean score of peer educators to be significantly higher. In the study conducted by Williams and Fowler, in which undergraduate paramedic and nursing students voluntarily chose to be peer educators [42], the end-of-year scores of the peer educators included in the study were significantly higher than their peers who were not in the study. Wong et al. [47], showed that thanks to the peer education program, peer educators got higher grades than the control group in the USMLE exam, which is required to be a doctor in America, and in the final exams of their faculties. While the mean of the peer educators’ exam was 43.22 points in our study, the other students’ exam mean was 33.78 (max point is 50.0). This difference between the two groups was statistically significant and is consistent with the literature results [37, 42, 47].

The mean scores of peer educators and students made a difference only in parasitology practices, and the groups were similar in all other mean scores. While this shows that the peer educators represent the universe in which they are chosen well, it shows that the benefit of the peer education model cannot be extended to other laboratory and theoretical courses.

## Conclusion

Peer education aims to increase class participation and success by reducing students’ stress levels. This educational model uses students’ social interactions with their peers. In this study, the peer education model was applied to medical students, and their satisfaction was presented. In this respect, the application of peer education will increase participation and interest in the lesson, and the students will feel more self-confident, inclined to teamwork, and have improved communication skills. In addition, in our study, exam performances were compared objectively. Although there is no significant difference compared with the previous years when we look at the class in general, the high exam success of peer educators who learn by teaching reveals the necessity of disseminating this education model. It will be beneficial to continue the peer education model in the following years, to compare the outputs with the existing data and expand it to other laboratory courses.

## Data Availability

All data generated or analysed during this study are included in this published article. The datasets used and analyzed during the current study are available from the corresponding author on reasonable request.

## Abbreviations

EPTO: The European Peer Training Organization
WHO: World Health Organization
